# Dosimetric impact of cylinder size in high‐dose rate vaginal cuff brachytherapy (VCBT) for primary endometrial cancer

**DOI:** 10.1120/jacmp.v17i5.6182

**Published:** 2016-09-08

**Authors:** Hualin Zhang, Mahesh Gopalakrishnan, Plato Lee, Zhuang Kang, Vythialingam Sathiaseelan

**Affiliations:** ^1^ Department of Radiation Oncology Robert H. Lurie Comprehensive Cancer Center, Northwestern University Feinberg School of Medicine, Northwestern Memorial Hospital Chicago IL USA

**Keywords:** ^192^Ir, Brachytherapy, high dose rate, endometrial cancer

## Abstract

The purpose of this study was to evaluate the dosimetric impact of cylinder size in high‐dose‐rate (HDR) vaginal cuff brachytherapy (VCBT). Sample plans of HDR VCBT in a list of cylinders ranging from 2.5 to 4 cm in diameter at 0.5 cm increment were created and analyzed. The doses were prescribed either at the 0.5 cm depth with 5.5 Gy for 4 fractions or at the cylinder surface with 8.8 Gy for 4 fractions, in various treatment lengths. A 0.5 cm shell volume called PTV_Eval was contoured for each plan and served as the target volume for dosimetric evaluation. The cumulative and differential dose volume histograms (c‐DVH and d‐DVH), mean doses (D‐mean) and the doses covering 90% (D90), 10% (D10), and 5% (D5) of PTV_Eval were calculated. In the 0.5 cm depth regimen, the DVH curves were found to have shifted toward the lower dose zone when a larger cylinder was used, but in the surface regimen the DVH curves shifted toward the higher dose zone as the cylinder size increased. The D‐means of the both regimens were between 6.9 and 7.8 Gy and dependent on the cylinder size but independent of the treatment length. A 0.5 cm variation of diameter could result in a 4% change of D‐mean. Average D90s were 5.7 (ranging from 5.6 to 5.8 Gy) and 6.1 Gy (from 5.7 to 6.4 Gy), respectively, for the 0.5 cm and surface regimens. Average D10 and D5 were 9.2 and 11 Gy, respectively, for the 0.5 cm depth regimen, and 8.9 and 9.7 Gy, respectively, for the surface regimen. D‐mean, D90, D10, and D5 for other prescription doses could be calculated from the lookup tables of this study. Results indicated that the cylinder size has moderate dosimetric impact, and that both regimens are comparable in dosimetric quality.

PACS number(s): 87.61.‐c, 87.53.Jw, 87.19.xj

## I. INTRODUCTION

The efficacy of intracavitary high‐dose‐rate (HDR) brachytherapy has been proven for decades.^1^, ^2^ Although multiple‐channel cylinder applicators are gaining popularity, the single‐channel HDR vaginal cuff brachytherapy (VCBT) still is a standard of care for early stage endometrial cancers in most radiation oncology clinics, either combined with external beam or delivered as a monotherapy of radiation.^(3)^ The primary reason is that it is simple, convenient, and almost foolproof. Clinical data indicated that the HDR VCBT not only provides a large dose boost to an endometrial lesion, but also reduces the risk of recurrence in the vagina and causes significantly less toxicity than pelvic external‐beam radiation therapy.^4^, ^5^, ^6^

Due to the highly elastic nature of the vagina, in the clinical practice of HDR VCBT, it is found that picking a cylinder with a 0.5 cm variation in diameter could easily become subjective. For example, a cylinder of 2.5‐cm in diameter could easily be chosen for a fitting simulation, but a cylinder of 3.0 cm in diameter may prove to be just optimal. Since the dose is prescribed either at 0.5 cm depth or at the cylinder/vaginal surface, different cylinder sizes mean different source‐to‐cylinder surface distances, and the HDR VCBT plan is normalized at either 0.5 cm depth or cylinder surface from the nonuniform brachytherapy dose distribution. Therefore, the doses received in the target volume are expected to be different for different cylinder applicators. In order to understand the potential dosimetric impact of cylinder size provided in the HDR VCBT, the dosimetric distributions of relevant target volumes in brachytherapy plans using various cylinder applicators need to be evaluated.

In this study, a series of HDR VCBT plans were generated by a treatment planning system (TPS) from a list of sample CT images with various sizes of cylinder applicators. Per our clinical guideline, two different fractionation regimens were planned for HDR VCBT: 1) 5.5 Gy prescribed to 0.5 cm depth delivered for 4 fractions, and 2) 8.8 Gy prescribed to the surface of the cylinder delivered for 4 fractions. The sizes of fraction doses were obtained from the ABS (American Brachytherapy Society) recommendations.^(7)^ The evaluation target volume was considered to be a 0.5 cm depth shell surrounding the cylinder with different treatment lengths. The rationale for using this target volume was based upon the literature,^(8)^ that reported approximately 95% of vaginal lymphatic channels (a potential surrogate for cancer cell location) are located within a 0.3 cm depth from the vaginal surface and that the density of lymphatic channels decreases with the depth.

## II. MATERIALS AND METHODS

### A. Clinical treatment plan and dose prescription

An HDR unit with an ^192^Ir source (Nucletron, Elekta AB, Stockholm, Sweden) was used for the HDR VCBT. A brachytherapy treatment planning system (TPS) (Oncentra, version 3.4, Elekta) was used to make the vaginal cuff brachytherapy plans utilizing cylinders (Nucletron, Elekta AB) with a range of diameters (2.5, 3.0, 3.5, 4.0 cm).

The two aforementioned fractionation regimens were alternatively used to generate the treatment plans. The sample CT images with various cylinder applicator sizes were scanned in 2‐mm slices and used to make CT image‐guided HDR VCBT plans.

**Figure 1:**
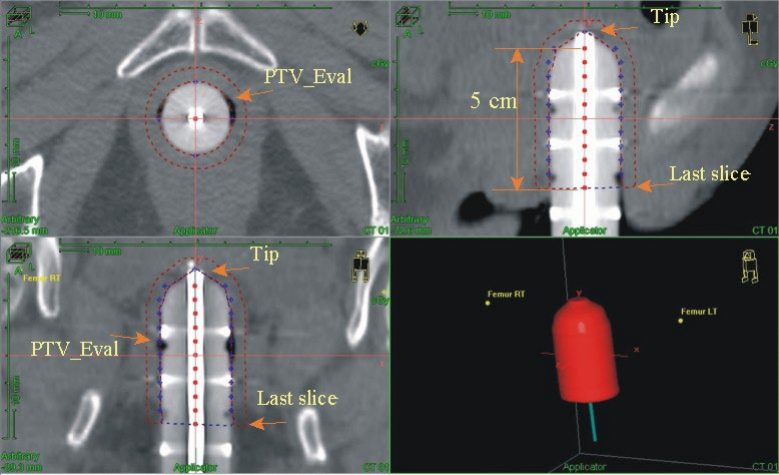
View of cylinder applicator and target volume PTV_Eval.

To determine the dwell positions of the source, the central thin metal tube of the cylinder applicator was first tracked in the TPS “Catheter Construction” workspace starting from the tip/edge of white bar and extended until 7 to 10 cm long, in the coronal or sagittal view with the “start from tip‐end” tracking option (Fig. 1). For the cylinder applicators involved in this study, the tip of the central metal tube is the tip of the applicator head. A tip space, the distance from the tip of the applicator to the first source dwell position (−6 mm) was experimentally determined and entered in the catheter construction. All other dwell positions were laid out after the first position with the 0.5 cm step size.

In this study, the HDR VCBT plans were generated as follows. A set of reference points were created at the prescription depth at each source dwell position. These “axis points” were used to normalize the prescription dose. Since the default step size was 0.5 cm, a 5‐cm treatment length, for example, had 11 dwell points (Fig. 1). In planning, the most proximal dwell point was not used to create a corresponding reference point, to avoid excessive extension of the prescription isodose line. The planning method of this study was the same as that used in another group's work.^(4)^ The cylindrical applicators used were composed of a combination of several discrete sections; the diameters of these sections decrease slightly at the junction regions to make them more identifiable on imaging. Using a nominal radius in creating these reference points has avoided these slight surface variations. In addition, the head of applicator is hemi‐ellipsoidal. The experimentally determined radii at different in‐head dwelling points were used to generate the corresponding reference points.

### B. Contouring the target volume

The volume of the cylinder was first contoured from the CT axial image and the nominal radius was used in the junction regions as well. The cylinder was made of plastic material and its CT number is not very different from water, so the image of the ellipsoidal applicator head was particularly fuzzy. One approach we used was to adjust and get the optimal/proper contras level by confirming the nominal diameter of cylinder from the middle section of the cylinder. In this way the uncertainty of contouring the plastic applicator head image was able to be reduced. The cylinder surface was contoured extending to the slice containing the last source dwell position, this volume was named Cylinder_TX. The Cylinder_TX was then expanded by 0.5 cm in all directions except for the inferior direction (Fig. 1). The margin was set 0 towards the inferior direction. This new volume was named Cylinder_exp_5mm. Another new volume called PTV_Eval was then made by subtracting Cylinder_TX from the Cylinder_exp_5mm: PTV_Eval = Cylinder_exp_5mm ‐ Cylinder_TX. PTV_Eval, a 0.5 cm thick shell volume, was used to serve as the target volume for dosimetric analysis for each plan.

It should be noted that so far the prescription point for HDR VCBT has been either at the cylinder surface or at a depth of 0.5 cm; other prescription depths have not been widely used. Although cancer cells may have different infiltration, it is possible the cancer may spread beyond 0.5 cm. However, because the dose curve of the HDR ^192^Ir source drops very fast with depth in tissue, if the prescription depth is greater than 0.5 cm — 1 cm, for example, adding 1.5 cm or 2 cm of cylinder radius, the surface dose would be inevitably very high for achieving a reasonable 1 cm depth prescription dose. Choo's study^(8)^ demonstrated that most cancer cells were located within 0.5 cm for their studied early‐stage endometrial cases; thus, a 0.5 cm shell target volume was used in this study. More regarding the HDR VCBT target volume is given in the discussion section.

### C. Comparing DVHs of PTV_Eval for different sizes of cylinders and different prescription regimens

Two prescription regimens were separately used to generate the plans with all available cylinder sizes and commonly used treatment lengths. The two prescription regimens are: 1) 5.5 Gy prescribed to a depth of 0.5 cm, also referenced as the 0.5 cm depth regimen, and 2) 8.8 Gy prescribed at the cylinder surface, also referenced as the surface regimen. For every treatment length, cylinder size, or prescription regimen, the cumulative dose‐volume histogram (c‐DVH) and differential dose‐volume histogram (d‐DVH) of PTV_Eval were separately exported from the TPS for dosimetric analysis. The work was repeated for the two prescription regimens. The D90 (dose covering 90% of PTV_Eval), D10 (dose covering 10% of PTV_Eval), and D5 (dose covering 5% of PTV_Eval) were obtained from the c‐DVH for each scenario. From the d‐DVH, the D‐mean (mean dose of PTV_Eval) was calculated for comparing the overall dosimetric quality.

## III. RESULTS

### A. DVHs of PTV_Eval

**Figure 2:**
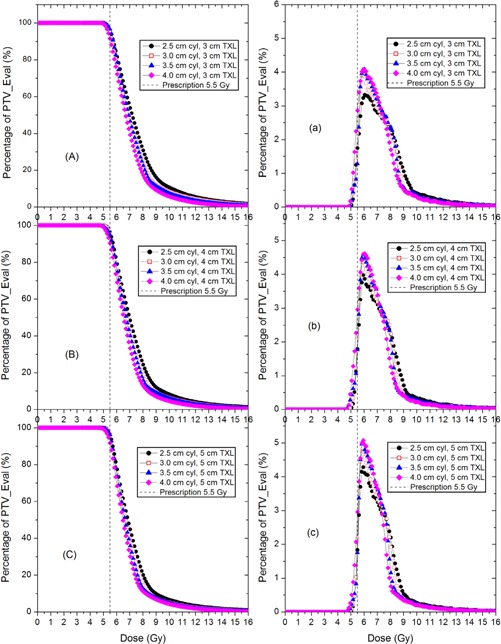
Cumulative (A, B, and C) and differential (a, b, c) DVH curves of PTV_Eval of the HDR VCBT plans using four sizes of cylinder applicators with three different treatment lengths. Prescription dose is 5.5 Gy, at a depth of 0.5 cm from the cylinder surface.

Figures 2(a) to (c) show the c‐DVHs of the PTV_Eval of the plans using four sizes of cylinders, at the treatment length of 3, 4, and 5 cm for the 0.5 cm depth regimen. As the cylinder size was increased from 2.5 cm to 4 cm, the c‐DVH curves were found to have shifted towards the lower dose zone. This dosimetric zone shift was also seen if the variable was treatment length. Figures 2(a) to (c) illustrate the d‐DVHs of the PTV_Eval. It was found that the dose peaks become steeper as the treatment length was increased from 3.0 to 5.0 cm. However, the most probable dose is ~ 6.5 Gy for all sizes of cylinders. The long tails of c‐DVH and d‐DVH curves are due to the tip/head surface region of applicator, which is the closest to the source dwell points, but the volume of the high dose zone (greater than 10 Gy) is very small (less than 0.5% of PTV_Eval).

**Figure 3:**
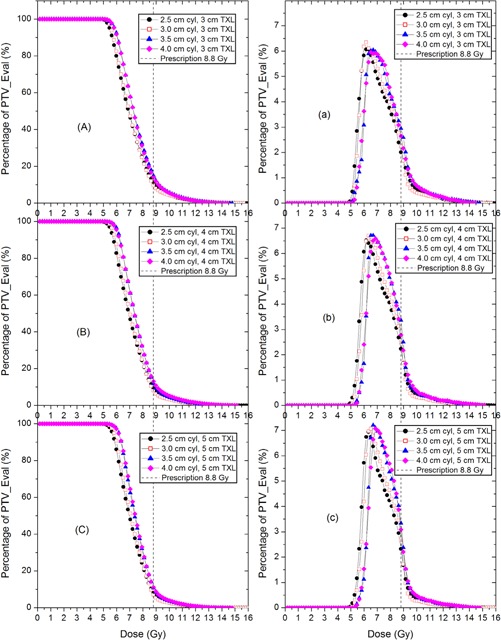
Cumulative (A,B and C) and differential (a, b, c) DVH curves of PTV_Eval of the HDR VCBT plans using four sizes of cylinder applicators with three different treatment lengths. Prescription dose is 8.8 Gy, at the cylinder surface.

Figures 3(a) to (c) show the c‐DVHs for the cylinder surface regimen. When the cylinder size is increased, the c‐DVH curves are found to have shifted towards the higher dose zone. The d‐DVHs (Figs. 3(a) to (c)) show that the dose spectrum has shifted towards the higher dose side when a larger cylinder size is used. The most probable dose was ∼7 Gy for all sizes of cylinders in this prescription regimen. The long tails of the c‐DVH and d‐DVH curves in each scenario are similar to those of the 0.5 cm depth regimen and are attributed to the cylinder tip/head surface region as well.

### B. D‐Mean, D90, D10, and D5 of PTV_Eval

Table 1 gives the D‐mean and D90 of different cylinder sizes for one fraction treatment as they relate to various treatment lengths and prescription regimens. The data in each scenario were the average of the three independent cases. For the 0.5 cm depth regimen, the D‐mean decreases with both the cylinder size and treatment length. For the cylinder surface regimen, however the D‐mean increases with the cylinder size but stays constant with the treatment length. The D‐mean is at least 130% of the prescribed dose for the 0.5 cm depth regimen, but only about 85% of the prescription dose for the surface regimen. A 0.5 cm variation of diameter could cause up to 4% change of D‐mean in the both regimens, whereas the D90 changes only by 2% for the same diameter change. In the 0.5 cm depth regimen, the D90 decreases only up to 3.6% (from 5.8 to 5.6 Gy) when the cylinder size is increased from 2.5 to 4.0 cm, and remains constant with the treatment length (from 3 to 5 cm) at a fixed diameter of cylinder. However, in the surface regimen, the D90 increased by 8% (from 5.9 to 6.4 Gy) with the cylinder size and 4% with the treatment length for the same geometrical changes.

For the readers who are using different prescription dose sizes, the D‐mean and D90 of their own regimens can be calculated by multiplying the prescription dose ratio with the corresponding value of Table 1. For example, if the prescription dose is 7 Gy/fraction at a depth of 0.5 cm for 3 fractions, the diameter of cylinder is 3.0 cm and the treatment length is 4 cm, the D‐mean and D90 for this regimen will respectively be 27.9 Gy (namely 7.3 Gy×1.27×3) and 21.7 Gy (5.7 Gy×1.27×3). The prescription dose ratio in this example is 7/5.5=1.27. The D‐mean and D90 of the 5.5 Gy regimen are 7.3 Gy and 5.7 Gy, respectively, taken from Table 1.

HDR brachytherapy delivers nonuniform dose to the target volume, so the high‐dose coverage needs to be analyzed and documented. Table 2 presents the D10 and D5 for different cylinder sizes, different treatment lengths, and different prescription regimens. It was found that the D10 and D5 decrease with the cylinder size in the 0.5 cm depth regimen, but increase with the cylinder size in the surface regimen. The average D10 is 9.0 Gy (ranging from 8.2 to 10.2 Gy) for the 0.5 cm depth regimen and 8.9 Gy (ranging from 8.6 to 9.2 Gy) for the surface regimen. The average D5s are 10.7 Gy (ranging from 9.7 to 12.3 Gy) and 9.7 Gy (ranging from 9.1 to 10.2 Gy), respectively, for the 0.5 cm depth and surface regimens. The D10 and D5 for other prescription dose sizes can also be calculated by using Table 2 following the same instruction used for calculating the D‐Mean and D90.

**Table 1:** D‐Mean and D90 of the PTV_Eval for four sizes of cylinders, three treatment lengths, and two prescriptions regimens. The data are from one fraction of treatment.

	*3 cm TXL*		*4 cm TXL*		*5 cm TXL*	
*Diameter (cm)*	*D‐Mean (Gy)*	*D90 (Gy)*	*D‐Mean (Gy)*	*D90 (Gy)*	*D‐Mean (Gy)*	*D90 (Gy)*
*Prescription: 5.5 Gy at 0.5 cm*						
2.5	7.8	5.8	7.5	5.7	7.3	5.7
3.0	7.5	5.7	7.3	5.7	7.1	5.7
3.5	7.4	5.7	7.2	5.7	7.0	5.7
4.0	7.1	5.6	6.9	5.6	6.9	5.6
*Prescription: 8.8 Gy at surface*						
2.5	7.3	5.7	7.3	5.8	7.3	5.9
3.0	7.3	5.7	7.3	5.9	7.4	6.0
3.5	7.6	6.1	7.6	6.2	7.7	6.4
4.0	7.6	6.1	7.7	6.2	7.7	6.4

TXL= the treatment length.

**Table 2:** D10 and D5 of the PTV_Eval for four sizes of cylinders, three treatment lengths, and two prescription regimens. The data are from one fraction of treatment.

	*3 cm TXL*		*4 cm TXL*		*5 cm TXL*	
*Diameter (cm)*	*D10 (Gy)*	*D5 (Gy)*	*D10 (Gy)*	*D5 (Gy)*	*D10 (Gy)*	*D5 (Gy)*
*Prescription: 5.5 Gy at 0.5 cm*						
2.5	10.2	12.3	9.5	11.4	9.0	10.8
3.0	9.5	11.7	8.9	10.9	8.4	10.1
3.5	9.3	11.1	8.8	10.6	8.4	10.0
4.0	8.9	10.3	8.4	9.8	8.2	9.7
*Prescription: 8.8 Gy at surface*						
2.5	8.9	9.7	8.7	9.4	8.6	9.1
3.0	9.0	9.7	8.7	9.4	8.6	9.2
3.5	9.2	10.2	8.9	9.8	8.9	9.5
4.0	9.2	10.2	9.1	10.1	8.9	9.7

TXL = the treatment length.

**Figure 4:**
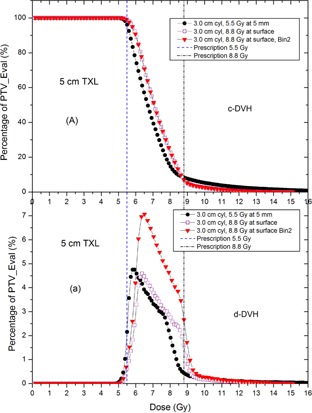
DVH curves of PTV_Eval for the same size of cylinder applicator (3.0 cm) and same treatment length (5 cm). The prescription doses are 5.5 Gy at the depth of 0.5 cm and 8.8 Gy at the cylinder surface, respectively. The 8.8 Gy regimen was also plotted in a larger bin size (Bin2) to show the potential hidden errors in d‐DVH comparison.

Figure 4 shows the DVH comparison between two prescription regimens at the same cylinder size (3.0 cm) and treatment length (5 cm). It was found that the c‐DVH and d‐DVH curves from two prescription regimens were similar but some noticeable differences were seen: a plan with the prescription of 8.8 Gy at the cylinder surface tends to create a slightly larger percentage of high dose regions than a plan with the prescription dose of 5.5 Gy at a depth of 0.5 cm. The

D‐means of two regimens (0.5 cm depth regimen vs. surface regimen) are within 3% difference (7.1 vs. 7.4 Gy), the D90s within 5% difference (5.7 vs. 6.0 Gy), D10s within 2% difference (8.4 vs. 8.6 Gy), D5s within 10% difference (10.1 vs. 9.2 Gy). The DVH curves with a larger bin size (Bin2) are also plotted in Fig. 4 for comparison. The potential error caused by using two different bin sizes in the d‐DVH comparison is discussed in the discussion section.

## IV. DISCUSSION

In this study, the dose distribution of HDR VCBT was assessed over a variety of cylinder diameters and lengths; the DVH, D‐mean, D90, D10, and D5 of the vaginal wall PTV_Eval were derived; and the dosimetric impact of cylinder size was evaluated.

When a choice is made to use the HDR VCBT in clinic, the primary merits considered in the selection are that the HDR VCBT can further decrease early stage endometrial cancer recurrence of standard whole pelvis EBRT.^(1)^ In addition, the HDR VCBT is comparable to pelvic EBRT for patients with high to intermediate risk of recurrence after total abdominal hysterectomy and bilateral salpingo‐oophorectomy, and the gastrointestinal toxicity is lower in HDR VCBT.^9^, ^10^, ^11^ The dosimetric spectrum (c‐DVH and d‐DVH) has revealed why such an advantage exists. The c‐DVH curves demonstrated that although the dose coverage curves do not drop as rapidly as a well‐planned IMRT and there exists a high dose tail, but the curves still drop quite steeply around 6 Gy; 90% of the PTV_Eval will receive at least 6 Gy, 50% of the PTV_Eval will receive 7 Gy, 10% receives 8.5 Gy, and about 3% receives up to 12 Gy (2, 3, 4). This implies a good target conformality and normal tissue sparing effect in the HDR VCBT. Because a significant amount of target (greater than 50%) will receive more than 28 Gy (7 Gy/fraction ×4 fractions) in the whole HDR VCBT course, and also because 10% of target volume with the largest tumor cell burden located at the surface^(8)^ could receive up to 34 Gy (8.5 Gy/fraction ×4 fractions), only 3.5% target volume — which also has the largest tumor burden — will receive 48 Gy (12 Gy/fraction ×4 fractions); therefore, the tumor control probability is expected to be high and the radiation complications are expected to be low.^(12)^ These results are supported by the clinical observations that a relatively low‐dose vaginal brachytherapy appears remarkably effective at eliminating the rate of vaginal recurrence, with low toxicity.^1^, ^9^, ^10^, ^11^

2, 3, 4 depict the d‐DVHs of different plans of two regimens, from which the dose spectra of the target volume can be revealed. All d‐DVH peaks are fairly narrow with the meaningful dose (irradiating more than 1% of the target volume) of the PTV_Eval ranging from 5.5 to 9 Gy. This would ensure that the HDR VCBT will achieve an intensive killing of cancer cells in the target volume while avoiding toxicity usually induced by a very large dose delivered to a large percent of volume in single‐fraction radiation. 3, 4 also demonstrate that a larger treatment length will produce a narrower d‐DVH peak implying that the dose distribution will become more uniform in the plan with a larger treatment length.

When the d‐DVHs were compared, an important lesson regarding the proper generation and comparison of the d‐DVHs between two different regimens was revealed. It was found that, in most TPS the number of the dose bins of the DVH was usually automatically taken from a convenient default number (50 or 100 for example), and the size of dose bin then was calculated based on the size of the prescription dose. Therefore, a larger size of prescription dose would have a larger bin size unless the bin number is manually increased. For example, an 8.8 Gy of prescription dose regimen will have a larger bin size than the 5.5 Gy regimen because the default bin number is the same. If one compares the c‐DVHs of the two prescription regimens, the results will be the same even if two different dose bin sizes are used (Fig. 4(a)). However, for a d‐DVH comparison, this may result in an erroneous conclusion if the bin sizes used in the two d‐DVHs are different. Figure 4(a) shows the difference of the d‐DVHs for the same plan (the plan of 8.8 Gy at surface) using two different bin sizes. When an automatically generated bin size (Bin2 in Fig. 4(a)) is used to generate the d‐DVH, the peak of the d‐DVH curve of the 8.8 Gy in the surface regimen is much bigger than that of the 5.5 Gy in the 0.5 cm regimen (which also used an automatically generated bin size, but its bin size is smaller since its prescription dose is smaller): the two regimens (8.8 Gy at surface vs 5.5 Gy at 0.5 cm depth) appear not comparable. However, if the bin number is manually adjusted in the TPS and so that the bin size of the d‐DVH of the 8.8 Gy regimen is changed to be the same as that used in the 5.5 Gy regimen, the d‐DVH curves of the both regimens (8.8 Gy at surface and 5.5 Gy at 0.5 cm depth) consequently show little difference. Therefore, in order to properly compare the d‐DVHs of two different regimens, the bin sizes of the two d‐DVHs should be the same.

A test was performed in the TPS for the 1 cm depth regimen. With a 3 cm diameter cylinder and 5 cm treatment length, it was found that the surface dose at the middle of cylinder (defined by the treatment length) was as much as 10.5 Gy for the 1 cm depth prescription compared to 7.8 Gy for the 0.5 cm depth prescription, for the same prescription dose of 5.5 Gy. At the middle surface of the applicator head, the dose from the 1 cm depth prescription was even as high as 16.80 Gy, compared to 11.20 Gy for the 0.5 cm depth prescription. The applicator tip point dose will be even higher, as reported in literature.^(12)^ This may partially explain why the prescription depth of the HDR VCBT is usually either at 0.5 cm depth or at the cylinder surface. Due to the rapid drop of the radial dose function of ^192^Ir HDR source with the depth, a depth prescription deeper than 0.5 cm will deliver too high of a dose at the vaginal surface, and thus more complications from radiation may occur.^(13)^

As reported in literature,^14^, ^15^ the HDR VCBT has some significant uncertainties. The uncertainties which existed in this study include source strength (2%); treatment planning (3%); medium dosimetry corrections (1%); volume contouring (3%); and dose delivery, including registration of applicator geometry to anatomy (5%), and interfraction/intrafraction changes between imaging and dose delivery (5%). If all of the uncertainty components are assumed to be not correlated, one can get the combined uncertainty of 8.5% (k=1) in the D‐mean, D90, D10, and D5 calculations using the quadrature calculation.

## V. CONCLUSIONS

The data analysis suggests that, for the HDR VCBT, the cylinder size has moderate and various impacts to the doses of the D‐mean, D90, D10, and D5 delivered in the target volume PTV_Eval, depending on the prescription regimen. A smaller diameter of cylinder will have a larger surface dose; a larger treatment length would provide a better dose uniformity. The D90 and D‐mean doses are, respectively, at least 102% and 130% of the prescribed dose for the 0.5 cm depth regimen, but are, respectively, only about 65% and 85% of prescription dose for the vaginal surface regimen. Both regimens tested in this study (5.5 Gy at the 0.5 cm depth and 8.8 Gy at the cylinder surface) are comparable.

## ACKNOWLEDGMENT

The authors thank Adam Prescott of Northwestern University for proofreading the manuscript.

## COPYRIGHT

This work is licensed under a Creative Commons Attribution 3.0 Unported License.
